# Smooth muscle cell‐specific *Tgfbr1* deficiency attenuates neointimal hyperplasia but promotes an undesired vascular phenotype for injured arteries

**DOI:** 10.14814/phy2.13056

**Published:** 2016-12-06

**Authors:** Mingmei Liao, Pu Yang, Fen Wang, Scott A. Berceli, Yasmin H. Ali, Kelvin L. Chan, Zhihua Jiang

**Affiliations:** ^1^ Division of Vascular Surgery and Endovascular TherapyUniversity of Florida College of MedicineGainesvilleFlorida; ^2^ Department of SurgeryCentral South University Xiangya HospitalChangshaHunanChina; ^3^ Malcom Randall VA Medical CenterGainesvilleFlorida

**Keywords:** Arterial restenosis, inducible gene deletion, receptor, transforming growth factor *β*, vascular phenotype

## Abstract

Neointimal hyperplasia (NIH) and inward wall remodeling cause arterial restenosis and failure of bypass vein grafts. Previous studies from our group suggest that transforming growth factor (TGF) *β* promotes these pathologies via regulating cell kinetics at the early stage and matrix metabolism at the late stage. Although these temporal TGF *β* effects may result from its signaling in different cell groups, the responsible cell type has not been identified. In the current study, we evaluated the effect of smooth muscle cell (SMC)‐specific TGF *β* signaling through its type I receptor TGFBR1 on NIH and wall remodeling of the injured femoral arteries (FAs). An inducible *Cre/loxP* system was employed to delete SMC *Tgfbr1* (*Tgfbr1* ^*iko*^). Mice not carrying the *Cre* allele (*Tgfbr1* ^*f/f*^) served as controls. The injured FAs were evaluated on d3, d7, and d28 postoperatively. *Tgfbr1* ^*iko*^ attenuated NIH by 92%, but had insignificant influence on arterial caliber when compared with *Tgfbr1* ^*f/f*^ controls on d28. This attenuation correlated with greater cellularity and reduced collagen content. Compared with *Tgfbr1* ^*f/f*^ FAs, however, *Tgfbr1* ^*iko*^ FAs exhibited persistent neointimal cell proliferation and cell apoptosis, with both events at a greater rate on d28. *Tgfbr1* ^*iko*^ FAs additionally contained fewer SMCs and more inflammatory infiltrates in the neointima and displayed a thicker adventitia than did *Tgfbr1* ^*f/f*^ FAs. More MMP9 proteins were detected in the adventitia of *Tgfbr1* ^*iko*^ FAs than in that of *Tgfbr1* ^*f/f*^ controls. Our results suggest that disruption of SMC *Tgfbr1* inhibits arterial NIH in the short term, but the overall vascular phenotype may not favor long‐term performance of the injured arteries.

## Introduction

Neointimal hyperplasia (NIH) and inward wall remodeling develop in stented arteries and vein bypass grafts and frequently cause failure of these revascularization procedures. Although cells derived from various lineages may contribute to these pathologies (Zalewski et al. 2002), smooth muscle cells (SMCs) are considered to be a major player in this process (Nguyen et al. 2013). In response to wall injury, SMCs lose their contractile property and acquire a synthetic phenotype that enables them to synthesize a plethora of growth factors, proinflammatory cytokines and chemokines, and proteases (Owens et al. 2004). These bioactive substances, in turn, act on SMCs themselves and other cell groups, amplifying the local inflammatory response (Bobik 2006). As a result, SMCs constantly proliferate and remodel wall structure, leading to restenosis or complete occlusion of the treated vessels (O’ Brien et al. 2011).

Transforming growth factor (TGF) *β* is one of the most potent phenotypic modulators for SMCs. At the developmental stage, it stimulates precursor cells to express a set of genes coding for contractile proteins, such as *α*‐actin, SM22, and SM myosin heavy chain, and guides them to an SMC lineage fate (Gomez and Owens 2012). The response of mature SMCs to TGF*β* is, however, context dependent. Under in vitro culture conditions, TGF*β* can be either mitogenic or proapoptotic, depending on cell density (Hneino et al. 2009). Cross‐talks to Wnt/*β*‐catenin (DiRenzo et al. 2016), VEGF (Shi et al. 2014), Notch (Blokzijl et al. 2003), and p38 (Seay et al. 2005) signaling pathways have been implicated as potential mechanisms for TGF*β* to produce these distinct biological outcomes. In mature vasculature, particularly the injured vessel wall, TGF*β* has been consistently documented to promote SMC proliferation, inhibit SMC apoptosis, and provoke vascular fibrosis (Bobik 2006). Accordingly, inhibition of TGF*β* activity using various strategies attenuated neointimal growth in animal models (Smith et al. 1999; Yamamoto et al. 2000; Lan et al. 2016). Translation of these experimental findings to clinical applications, however, has been of limited success. The PRESTO trial, for example, reported that inhibition of TGF*β* with Tranilast failed to reduce the incidence of restenosis of stented coronary arteries (Holmes et al. 2002). Nevertheless, next‐generation drugs with more specific and powerful blocking of various TGF*β* signaling pathways have been recently developed and have advanced to clinical trials in the field of oncology (Neuzillet et al. 2015). Although these anti‐TGF*β* therapeutics are available in the market, the response of injured vessels to TGF*β* blockade has not been fully characterized. A detailed characterization of the resultant vascular phenotype will help tailor available anti‐TGF*β* therapeutics to maximize their benefits and minimize the potential side effects in the target vessels.

Previous studies from our laboratory and other groups suggest that TGF*β* promotes NIH and vascular remodeling at different stages via different mechanisms. At the early stage, TGF*β* promotes NIH via its mitogenic and profibrotic functions (Bobik 2006). We have previously shown that TGF*β* enhances the recruitment of fibroblasts to the adventitial layer, contributing to early inward wall remodeling (Jiang et al. 2007b). As neointimal growth progresses to a more advanced stage, TGF*β* functions primarily as a profibrotic factor, due to an increased ratio of TGFBR1 to TGFBR2 production in neointimal cells (McCaffrey 2000; Jiang et al. 2009). In the vessel wall, all vascular cells are fully equipped with TGF*β* signaling components and could potentially serve as cellular effectors for TGF*β* (Pardali and Ten 2012). While SMCs and fibroblasts are generally accepted as the major TGF*β* responders (Khan et al. 2007; Siow and Churchman 2007), an elegant recent study added endothelial cells to the list by demonstrating a significant contribution of TGF*β*‐mediated endothelium–mesenchymal transition to neointimal thickening (Cooley BC et al. 2014). TGF*β* may also benefit the injured vessels, however, by promoting endothelial cell proliferation and thus repairing the denuded luminal surface (Goumans et al. 2009). These temporal and cell‐type‐specific TGF*β* issues must be addressed prior to advancing existing anti‐TGF*β* treatments to prevent vascular restenosis. In addition, previous preclinical studies of anti‐TGF*β* therapies focused primarily on neointimal thickening, with less attention being paid to other phenotypic traits, such as SMC differentiation and status of local inflammation (Wolf et al. 1994; Kingston et al. 2001; Ryan et al. 2003; Heaton et al. 2008). Finally, recent studies (Li et al. 2014; Hu et al. 2015), including our previous study (Schmit et al. 2015), have demonstrated that an acute loss of SMC TGF*β* breaks aortic wall homeostasis and induces spontaneous aortic aneurysm formation. Although the peripheral arteries, including the common carotid and femoral arteries, appear to be unaffected, it raises concern about whether or not fully blocking TGF*β* benefits or harms the remodeling process of injured arteries. To address these critical issues, the current study evaluated the effect of SMC‐specific TGF*β* type I receptor deficiency on NIH, geometric remodeling, and local inflammatory response in injured arteries.

## Methods

### Experimental animals

This study performs within the guidelines of the National Institute of Health Guide for the Care and Use of Laboratory Animals. All animal surgeries were approved by the Institutional Animal Care and Use Committee of the University of Florida. Smooth muscle cell‐specific *Tgfbr1* deletion (*Tgfbr1* ^*iko*^) was achieved via an inducible *Cre/loxP* system driven by a *Myh11* promoter as we have previously described (Schmit et al. 2015). Briefly, the *Tgfbr1* ^*f/f*^ strain was crossed with the *Myh11‐CreER* ^*T2*^ strain carrying the *Cre* allele on the Y chromosome (Wirth et al. 2008). Offspring littermates were genotyped to screen for *Tgfbr1* ^*f/f*^.*Myh11‐CreER* ^*T2+/*^ male mice. In our breeding experiments with the *Tgfbr1* ^*f/f*^.*Myh11‐CreER* ^*T2+/*^ strain, we identified and established a colony that carries the *Myh11‐CreER* ^*T2*^ allele on the X chromosome (details of this strain will be reported in a separate manuscript). With this colony, we were able to produce male mice not carrying the *Cre* allele (*Tgfbr1* ^*f/f*^ *.Myh11‐CreER* ^*T20/*^) for use as controls in this study. Male mice at 10–11 weeks of age were used in the study and were all on a C57BL/6 background. *Tgfbr1* ^*f/f*^.*Myh11‐CreER* ^*T2+/*^ mice were treated with tamoxifen via intraperitoneal injections (2.5 mg/mouse per day for five consecutive days; Sigma, St. Louis, MO; T5648) to induce *Tgfbr1* ^*iko*^. Animals in the control groups received the same treatment. Using this protocol, we consistently achieved a recombinant efficiency of greater than 90% in vascular SMCs of mice carrying the *Myh11‐CreER* ^*T2*^ allele (Yang et al. 2016).

### Experimental model and sample collection

On the day following the last tamoxifen injection (designated as day 0, d0), the right femoral artery (FA) of the injected mice was denuded of endothelium and mechanically distended via passing a metal guide wire (outside diameter 0.381 mm) through the lumen for about 5 mm proximal to the wire insertion (Yang et al. 2016). All animals received a single dose of bromodeoxyuridine (BrdU, 50 mg/kg, Thermo Fisher, Waltham, MA; 000103) via intraperitoneal injection 24 h prior to the scheduled sample collection. Surgical samples were harvested at d3, d7, or d28 postoperatively (*n *= 6–7 per group of either genotype). The distal end of each specimen was incised at a site 1–1.5 mm proximal to the wire insertion to avoid a foreign‐body response to the ligature placed to close the wire insertion. The proximal end of each sample was labeled with a black silk suture. Uninjured *Tgfbr1* ^*f/f*^ (*n *= 5) and *Tgfbr1* ^*iko*^ FAs (*n *= 5) were collected on d0 and served as the baseline reference for the subsequent morphological and cellular analyses. Both *Tgfbr1* ^*iko*^ (Schmit et al. 2015) and *Tgfbr2* ^*iko*^ (Li et al. 2014; Hu et al. 2015) have been found to cause spontaneous aortic aneurysm formation. Although a phenotype with an acute loss of TGF*β* signaling has never been observed for FAs on gross examinations, it was unclear whether or not *Tgfbr1* ^*iko*^ caused histologically evident structural defects in those peripheral arteries not exposed to wire injury. To assess the injury‐independent effect of *Tgfbr1* ^*iko*^ on the structural integrity of FAs, we collected another set of uninjured *Tgfbr1* ^*iko*^ FAs (*n *= 4) on d13 (when *Tgfbr1* ^*iko*^ aortas typically develop observable histological defects in the ascending segments) and compared them with age‐matched and tamoxifen‐treated *Tgfbr1* ^*f/f*^ FAs (*n *= 4). To match the anatomic location of the injured FAs, only the right FAs were assayed in all experiments. All samples were perfusion fixed with 10% neutral buffered formalin and paraffin embedded in an orientation that assured the subsequent sectioning would begin from the distal end of the specimen.

### Histology and morphometry

A set of cross‐sections (5.0 *μ*m) were collected from locations 0, 200, and 400 *μ*m from the distal end of the FA samples, at the site where a complete cross‐section was first observed during the serial sectioning, a point defined as the “0 *μ*m.” This set of sections was stained using Masson's trichrome staining protocol for histologic evaluation and morphometric measurements. A total of five or six sets of sections at the 200 *μ*m location were collected for each FA sample and saved for other assays carried out in this study. In the mouse FA wire injury model, a significant number of cells from various sources may home to the injured vessel wall, which results in the neointima encompassing a mixed cell population (Tanaka et al. 2003; Nguyen et al. 2013). In addition, because of fusion of the neointimal with the medial layers, we were unable to confidently determine the border between these anatomical layers. The neointima and media were therefore treated as a combined single layer during data acquirement and termed as the “myointima” in this study. Using digital imaging software (Zen lite 2012, Zeiss, Peabody, MA), we traced the luminal surface and external elastic lamina (EEL) to obtain luminal area, area within the EEL, and lengths of the circumferences on the Masson's staining images. With these measurements, we calculated the thickness of the myointimal layer for each specimen. An average thickness of the three indicated locations was calculated to represent myointimal hyperplasia in each FA sample and entered for statistical analysis. Detailed methods of morphometry, as well as Movat's staining, have been described in our previous studies (Jiang et al. 2004, 2009; Yang et al. 2016).

### Picrosirius red staining

Paraffin sections were dewaxed, rehydrated, and stained with picrosirius red solution (0.1% sirius red in saturated picric acid) for 1 h. Specimens were then washed with acidified water (0.5% acetic acid, v/v), dehydrated, and then mounted on coverslips. Assays were evaluated under polarized light. Fibers illuminated in green, yellow, and red were considered to be collagens and the color intensity of these fibers was measured on a gray scale using Zen lite 2012 software (Zeiss). Collagen content in the myointimal and adventitial layers was expressed as an intensity (arbitrary units), normalized to the area of interest.

### TUNEL staining

Apoptotic cells in the injured FAs were identified using terminal deoxynucleotidyl transferase‐mediated dUTP nick end‐labeling (TUNEL) assays (Roche, Indianapolis, IN; 11684795910) as described previously (Jiang et al. 2007a,b). Cell nuclei were counterstained using propidium iodide (Sigma, P4170). Uninjured *Tgfbr1* ^*f/f*^ FAs were included in the assays as biological negative controls. Assays were evaluated with fluorescent microscopy to count TUNEL^+^ and total nuclei within the myointimal layer. Data were expressed as the percentage of TUNEL^+^ nuclei.

### Immunohistochemistry and immunofluorescence staining

Cell proliferation in the injured FAs was evaluated with immunohistochemistry (IHC) staining of BrdU incorporation, as we have detailed previously (Jiang et al. 2009). Uninjured *Tgfbr1* ^*f/f*^ FAs were included in the assays as biological negative controls. Briefly, antigens were unmasked with a citrate‐based antigen retrieval buffer (Vector Labs, Burlingame, CA; H‐3300) and detected with a rat anti‐BrdU primary antibody (Serotec, Raleigh, NC; MCA2060) and a rabbit anti‐rat secondary antibody (Vector Labs, BA‐4001). Assays were developed with an ABC (Vector Labs, PK6100) and a DAB kit (Vector Labs, SK4100). Nuclei were counterstained with hematoxylin, and the BrdU‐positive and total nuclei within the myointimal layer were counted. Data were expressed as percentage of the BrdU^+^ nuclei.

Leukocyte infiltrates were detected using an antibody against the pan leukocyte antigen CD45 (1: 100, Rat Anti‐Mouse IgG2b, BD Pharmingen, San Jose, CA; 30‐F11). The levels of MMP2 and MMP9 were assessed using a goat anti‐MMP2 (1:100, R&D system, Minneapolis, MN; AF1488) and a goat anti‐MMP9 (1:100, R&D system, AF909), respectively, to estimate the production of these proteases. Secondary antibodies applied to these assays include an Alexa Fluor 488 donkey anti‐goat IgG (1: 200, Life Technologies, Carlsbad, CA; A11055) and an Alexa Fluor 546 goat anti‐rat IgG (1: 200, Life Technologies, A11081). Cell nuclei were labeled with DAPI (Sigma, D9542). Sections treated with rat isotype IgG2b (1: 100, R&D system, MAB0061), blocking buffer, or nonimmunized rabbit IgG (1: 100, Novus, Littleton, CO; NBP2‐24891) were used as a negative control. All assays were evaluated under a fluorescent microscope. Monochrome images were acquired and analyzed using Zen lite 2012 software (Zeiss) to quantify specific staining signals in the myointimal and adventitial layers. Data of CD45^+^ cells were expressed as the percentage of CD45^+^ area in each layer, whereas data of MMP2‐ and MMP9‐positive staining were calculated as a normalized intensity of the specific signals in each layer. The adventitial layer was defined as the region between EEL and the outermost edge of the dense perivascular tissue.

### Statistical analysis

All data are expressed as the mean ± SEM. Statistical analyses were performed using Sigma Plot 13.0. (San Jose, CA) Datasets were evaluated using normality and equivalence variance testing. For those failing this evaluation, logarithmic and exponential transformations were employed to meet these requirements. Student's *t*‐test was used, when appropriate, for comparison between two groups, whereas two‐way analysis of variance (ANOVA) and two‐way repeated measures ANOVA were performed for comparisons between genotypes and anatomic layers, respectively, with Holm–Sidak pairwise multiple comparison analysis being used for post hoc tests. *P* < 0.05 was considered statistically significant.

## Results

### *Tgfbr1* ^*iko*^ is well tolerated by the uninjured FAs

**Figure 1:**
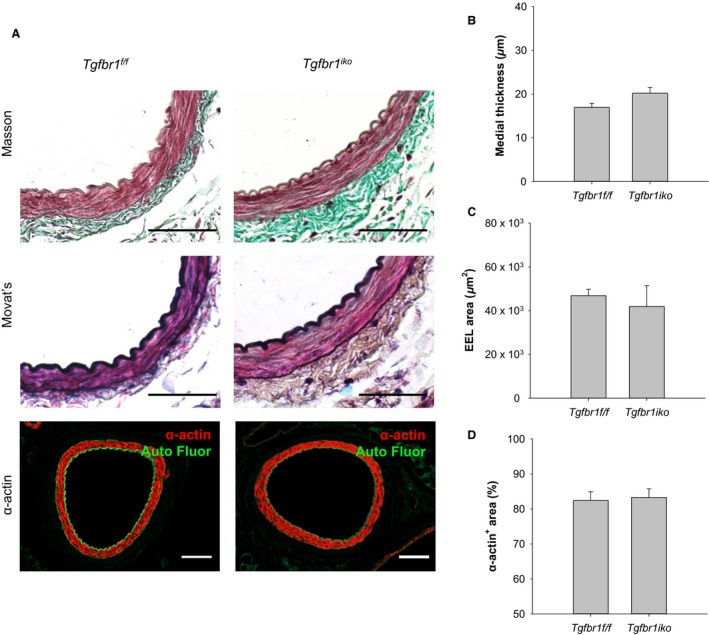
*Tgfbr1* ^*iko*^ is well tolerated by uninjured femoral arteries (FAs). (A) Masson's, Movat's, and anti‐*α*‐actin staining of uninjured *Tgfbr1* ^*f/f*^ and *Tgfbr1* ^*iko*^ FAs (*n *= 4, per group) on d13. Scale bars, 50 *μ*m. Medial thickness (B), EEL area (C), and percentage of *α*‐actin^+^ area in the medial layer of the indicated FAs (D).

We have previously reported that *Tgfbr1* ^*iko*^ aortas develop spontaneous aortic aneurysms, with the structural defects becoming evident on d13 (Schmit et al. 2015). In this study, we compared the histology of uninjured *Tgfbr1* ^*iko*^ and *Tgfbr1* ^*f/f*^ FAs at the same time point. Consistent with our previous experience with these strains, gross pathology was not noted for FAs of either genotype under a dissecting microscope. For both *Tgfbr1* ^*iko*^ and *Tgfbr1* ^*f/f*^ FAs (Fig. 1A), Masson's and Movat's staining revealed a well‐organized medial structure with intact internal and external elastic laminae (IEL and EEL, respectively) bracketing layers of SMCs. The adventitial layer comprised loose connective tissue rich in collagens. Cells located in the tunica media of FAs of either genotype were homogeneously *α*‐actin positive, indicating a differentiated contractile phenotype for these SMCs. Overall, *Tgfbr1* ^*iko*^ FAs were indistinguishable from *Tgfbr1* ^*f/f*^ FAs when evaluated with the measurements of EEL area (*P *= 0.621), medial thickness (*P *= 0.857), and fraction of *α*‐actin cells (*P *= 0.812) in the tunica media (Fig. 1B–D).

### Ablation of SMC‐specific *Tgfbr1* inhibits myointimal hyperplasia but promotes intense inflammatory response in injured femoral arteries

**Figure 2:**
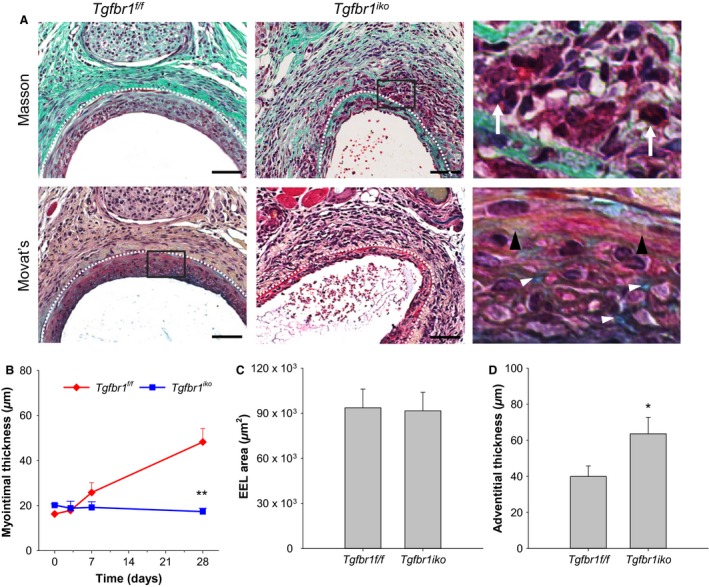
*Tgfbr1* ^*iko*^ inhibits hyperplastic myointimal thickening in injured femoral arteries (FAs). (A) Masson's and Movat's staining of injured *Tgfbr1* ^*f/f*^ and *Tgfbr1* ^*iko*^ FAs. Magnified view of the boxed area is shown on the far right. White dashed lines delineate external elastic lamina (EEL). White arrows point to inflammatory infiltrates, while black and white diamonds indicate deposition of collagen (yellow) and proteoglycans (blue), respectively. Scale bars, 50 *μ*m. Myointimal thickening (B), EEL area (C), and adventitial thickness (D) in *Tgfbr1* ^*f/f*^ and *Tgfbr1* ^*iko*^ FAs (*n *= 6–7 in each group). **P = 0.008, two‐way ANOVA (B); *P *= 0.908, *t‐*test (C); *P = 0.041, *t‐*test (D).

The metal wire‐mediated intramural injury induced robust hyperplastic neointimal thickening in *Tgfbr1* ^*f/f*^ FAs. Both Masson's and Movat's staining revealed that the injured arterial wall had remodeled to two layers by d28 (Fig. 2A, left column). The tunica media fused with the neointimal tissue, becoming a single myointimal layer where cells were highly disorganized, and the extracellular matrix comprised abundant collagen (yellow) and proteoglycans (blue). The adventitia displayed histological features typical of chronic fibrosis, including rich collagen deposition and a few inflammatory infiltrates (defined as polymorphonuclear or mononuclear cells with a rounded shape) located among fibroblasts. In contrast, *Tgfbr1* ^*iko*^ FAs formed only a thin layer of neointima when examined on d28. At this time point, the residual tunica media displayed a disrupted structure with infiltration of inflammatory cells. In the adventitial layer, *Tgfbr1* ^*iko*^ FAs exhibited a higher cellular density and contained much more inflammatory infiltrates compared to *Tgfbr1* ^*f/f*^ FAs (Fig. 2A, right column). Over the course of the hyperplastic response, myointima was readily detectable in *Tgfbr1* ^*f/f*^ FAs 7 days after injury, followed by a progressive myointimal thickening, whereas changes in myointimal thickness were insignificant in *Tgfbr1* ^*iko*^ FAs. As a result, *Tgfbr1* ^*iko*^ FAs developed a significantly thinner myointima than did *Tgfbr1* ^*f/f*^ FAs (17.4 ± 1.4 *μ*m vs. 48.2 ± 6.0 *μ*m, *P *< 0.001; Fig. 2B) by d28. The EEL area of the injured FAs was similar between the two groups (Fig. 2C). *Tgfbr1* ^*iko*^ FAs formed a much thicker layer of adventitia, however, compared to *Tgfbr1* ^*f/f*^ FAs (Fig. 2D), indicating that the impact of *Tgfbr1* ^*iko*^ on geometric remodeling of the injured arterial wall is more pronounced in the adventitia. These histological disparities led us to further characterize the cell kinetics and the resultant phenotypes of the injured FAs.

### *Tgfbr1* ^*iko*^ enhances both proliferation and apoptosis of the myointimal cells

**Figure 3:**
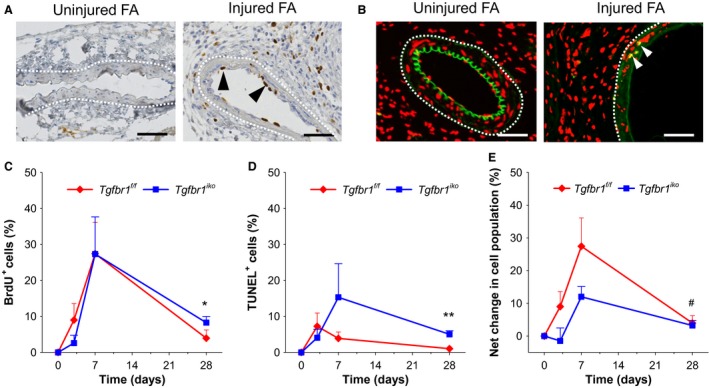
*Tgfbr1* ^*iko*^ accelerates myointimal cell proliferation and apoptosis. Specificity of the anti‐BrdU (A) and TUNEL (B) labeling assays evaluated on *Tgfbr1* ^*f/f*^ femoral arteries (FAs) with the indicated manipulation on d7. Black arrowheads: BrdU^+^ cells (brown); white arrowheads: TUNEL ^+^ cells (yellow); white dash lines: EEL; blue and red: nuclear counterstain; and green: autofluorescence. Scale bars, 50 *μ*m. Note that BrdU^+^ cells and TUNEL ^+^ cells were detected only in the injured FAs. Quantitative analysis of the BrdU^+^ cells (C), TUNEL ^+^ cells (D), and the net change of cell population (E) in the myointimal layer (*n* = 5–7 per group of either genotype at each time point). Time: *P *< 0.001 (two‐way ANOVA) for all three measured variables. *P = 0.037 (C), **P = 0.003 (D), and ^#^ *P =* 0.834 (E) by *t‐*tests.

Proliferation and apoptosis of the myointimal cells were evaluated using anti‐BrdU and TUNEL staining assays, respectively. Uninjured *Tgfbr1* ^*f/f*^ FAs were utilized as biological negative controls to validate the assay protocols (Fig. 3A and B). Under the optimized conditions, cells stained positive for BrdU incorporation or TUNEL were not detected in the tunica media of uninjured FAs, but were present in the injured *Tgfbr1* ^*f/f*^ FAs collected on d7. With the validated protocols, we quantified the temporal changes in the rate of proliferation and apoptosis for myointimal cells in *Tgfbr1* ^*f/f*^ and *Tgfbr1* ^*iko*^ FAs. The proliferation of myointimal cells peaked at d7, with a similar rate reached by both groups, although *Tgfbr1* ^*iko*^ FAs subsequently experienced a slower returning pace than *Tgfbr1* ^*f/f*^ FAs. As a result, a significantly higher rate of proliferation was detected in *Tgfbr1* ^*iko*^ FAs than in *Tgfbr1* ^*f/f*^ FAs on d28 (*P *= 0.037; Fig. 3C). A differential temporal pattern of cell apoptosis was also found between *Tgfbr1* ^*iko*^ and *Tgfbr1* ^*f/f*^ FAs. In *Tgfbr1* ^*f/f*^ FAs, cell apoptosis reached a low plateau on d3, followed by a sharp return to baseline level. In contrast, the rate of apoptosis in *Tgfbr1* ^*iko*^ FAs continued increasing until d7. Although the rate decreased thereafter, it remained significantly greater than that estimated for *Tgfbr1* ^*f/f*^ FAs (*P *= 0.003; Fig. 3D). The net change in cell population was similar between the two groups when evaluated at all indicated time points, however, suggesting that loss of SMC‐*Tgfbr1* accelerated myointimal cell kinetics, with a proportional increase in both cell proliferation and apoptosis.

### *Tgfbr1* ^*iko*^ attenuates collagen accumulation in the hyperplastic myointimal lesions

**Figure 4:**
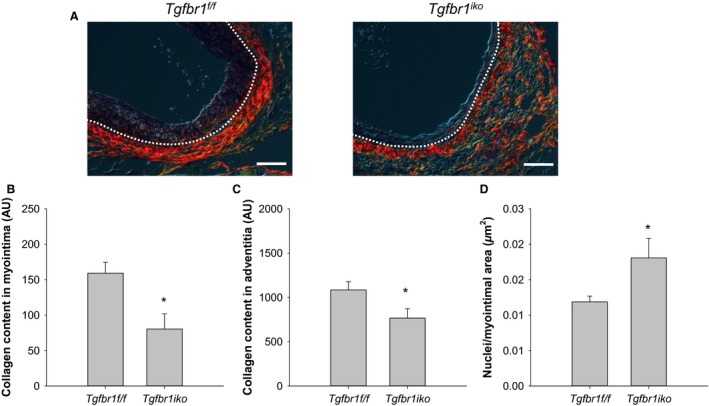
*Tgfbr1* ^*iko*^ attenuates deposition and assembly of collagen fibers in both myointimal and adventitial layers. (A) Sirius red staining of *Tgfbr1* ^*f/f*^ and *Tgfbr1* ^*iko*^ FAs (d28, *n* = 7 per genotype) evaluated under polarized light. White dashed lines delineate EEL. Red and yellow: thick collagen fibers; Green: thin collagen fibers. Scale bars, 50 *μ*m. Total collagen content in the myointimal (B) and adventitial (C) layers of FAs with the indicated genotype. Data were expressed as a normalized intensity in an arbitrary unit (AU). *P = 0.013 (B), *P = 0.047 (C), *t‐*test. (D) Cell density in the myointimal lesion of FAs with the indicated genotype. *P = 0.033, *t‐*test.

In addition to cellular expansion, matrix accumulation, particularly collagen deposition, is another major contributor for myointimal expansion. We therefore evaluated collagen content in *Tgfbr1* ^*iko*^ and *Tgfbr1* ^*f/f*^ FAs using sirius red staining. *Tgfbr1* ^*f/f*^ FAs contained abundant thin (greenish color) and a few thick (yellow and red) collagen fibers in the myointimal layer, with thick collagen fibers (yellow and orange‐red) densely assembled in the adventitia (Fig. 4A). In contrast, *Tgfbr1* ^*iko*^ FAs contained only a few thin but no thick collagen fibers in the myointima. In the adventitia, thick collagen fibers were loosely distributed, with the space filled by thin collagen fibers. Quantitatively, *Tgfbr1* ^*iko*^ FAs contained significantly less collagen in both myointimal (*P *= 0.013; Fig. 4B) and adventitial (*P *= 0.047; Fig. 4C) layers than did *Tgfbr1* ^*f/f*^ FAs. As a result, myointimal cells were more densely packed in *Tgfbr1* ^*iko*^ FAs than in *Tgfbr1* ^*f/f*^ FAs (*P *= 0.033; Fig. 4D).

### *Tgfbr1* ^*iko*^ FAs contain fewer α‐actin‐positive cells than *Tgfbr1* ^*f/f*^ FAs

Our data showed that less myointima formed in *Tgfbr1* ^*iko*^ FAs than in *Tgfbr1* ^*f/f*^ FAs after intramural injury (Fig. 2).It has been recognized, however, that the “healthy status” of the vessel wall and the hyperplastic myointimal thickening are equally important for long‐term performance of the treated vessels (Ryomoto et al. 2002). We therefore characterized other phenotypic traits for the injured *Tgfbr1* ^*f/f*^ and *Tgfbr1* ^*iko*^ FAs 28 days after the initiation of myointimal thickening.

**Figure 5:**
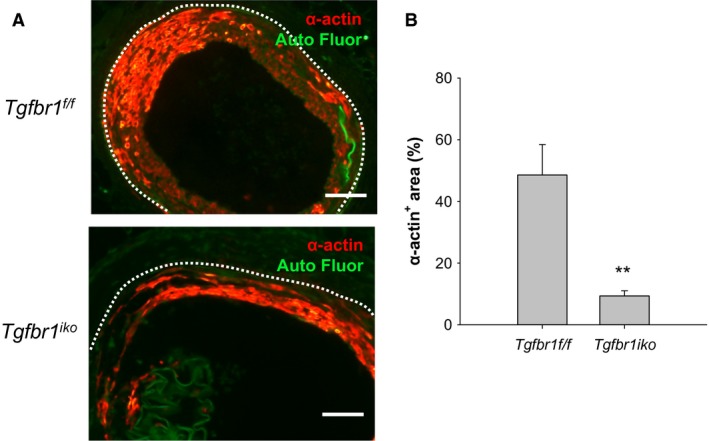
*Tgfbr1* ^*iko*^ femoral arteries (FAs) contain fewer *α*‐actin‐producing cells than do *Tgfbr1* ^*f/f*^ FAs. (A) Immunofluorescence staining of *α*‐actin. Cells with *α*‐actin production were labeled in red. Green: autofluorescence; white dashed lines: EEL. Scale bars, 50 *μ*m. (B) Content of *α*‐actin^+^ cells in the myointimal layer 28 days after injury (*n* = 7 per genotype). Data were expressed as percentage of *α*‐actin^+^ area in the corresponding myointimal region. **P = 0.002, *t‐*test.

We first quantified the content of contractile SMCs in these FAs. During myointimal hyperplasia, medial SMCs migrate to the tunica intima, where these cells undergo dedifferentiation at the early stage and reacquire a contractile phenotype (i.e., differentiation) at the advanced stage (Owens et al. 2004). By using immunofluorescence assays, we measured production of SM *α*‐actin, one of the major contractile markers that can be reacquired by dedifferentiated SMCs (Owens et al. 2004), in the myointimal layer of *Tgfbr1* ^*f/f*^ and *Tgfbr1* ^*iko*^ FAs. Figure 5A shows staining of *α*‐actin (red) in these FAs. The vast majority of myointimal cells in *Tgfbr1* ^*f/f*^ FAs produced *α*‐actin, whereas a large portion of the myointimal cells in *Tgfbr1* ^*iko*^ FAs were unable to produce *α*‐actin at this time point. Quantitatively, *Tgfbr1* ^*iko*^ FAs contained approximately four times fewer *α*‐actin‐positive cells than did *Tgfbr1* ^*f/f*^ FAs in the myointimal tissue (*P *= 0.002; Fig. 5B).

### *Tgfbr1* ^*iko*^ exacerbates accumulation of inflammatory infiltrates in the FA lesions

**Figure 6:**
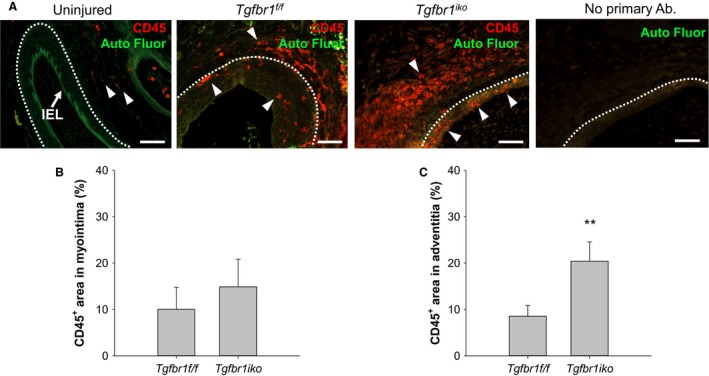
*Tgfbr1* ^*iko*^ femoral arteries (FAs) enclose more inflammatory infiltrates than do *Tgfbr1* ^*f/f*^ FAs at the advanced stage. (A) Immunofluorescence staining of CD45 in 28‐day‐old FA lesions. An uninjured FA was included as a biological negative control (i.e., no CD45 signal in the tunica media) in the assays. A panel of “no primary antibody” negative control is shown on the far right. Red: CD45^+^ cells (arrowheads); green: autofluorescence; white dashed lines: EEL. Scale bar, 50 *μ*m. Fraction of CD45^+^ area in the myointima (B) and adventitia (C) 28 days after injury (*n* = 7 per genotype). *P *> 0.05 (B), **P = 0.009 (C), *t‐*test.

In addition to the phenotypic switching of SMCs, chronic inflammation is another determinant of the long‐term performance of arteries which are reopened through interventional procedures (Newby and Zaltsman 2000). To evaluate the impact of *Tgfbr1* ^*iko*^ on the inflammatory status of established myointimal lesions, we compared the content of CD45 (a pan leukocyte marker)‐positive cells in d28 *Tgfbr1* ^*f/f*^ and *Tgfbr1* ^*iko*^ FAs. CD45‐positive cells were detected only sparsely in the tunica adventitia, but not noted in the tunica intima or media of uninjured FAs, indicating a specific labeling of leukocytes by the anti‐CD45 antibody applied to the assays (Fig. 6A). In *Tgfbr1* ^*f/f*^ FAs, CD45 positive inflammatory cells were scarcely scattered in the myointimal layer and occasionally present as small clusters in the adventitia. In contrast, *Tgfbr1* ^*iko*^ FAs displayed an intense infiltration of CD45 positive cells in both myointimal and adventitial layers. Although the difference in CD45‐positive cell content in the myointimal layer was not statistically significant (*P *> 0.05) between the two groups, the quantitative data indicated a trend of enhanced inflammatory cell accumulation in *Tgfbr1* ^*iko*^ FAs compared to *Tgfbr1* ^*f/f*^ FAs (Fig. 6B). The difference between the two groups was more pronounced in the adventitia, with the number of CD45 positive cells in *Tgfbr1* ^*iko*^ FAs being twice as high as that in *Tgfbr1* ^*f/f*^ FAs (*P *= 0.009; Fig. 6C).

### *Tgfbr1* ^*iko*^ leads to an enhanced production of MMP9 in adventitia of the injured FAs

**Figure 7:**
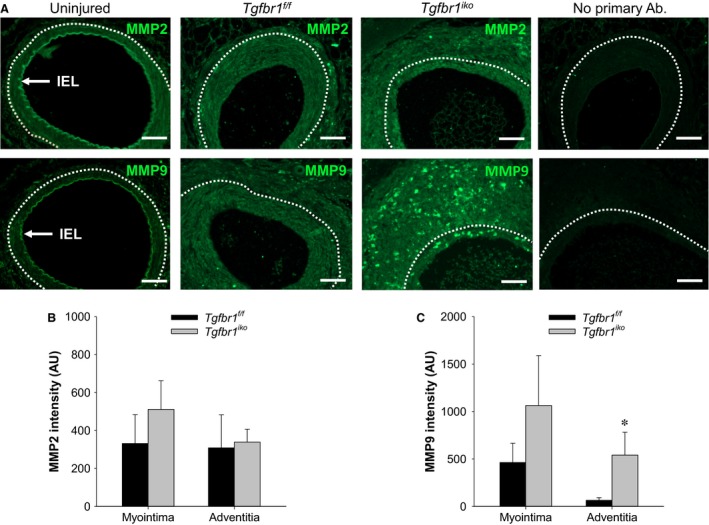
*Tgfbr1* ^*iko*^ femoral arteries (FAs) produce higher levels of MMP9 than do *Tgfbr1* ^*f/f*^ FAs in the adventitial layer at the advanced stage. (A) Immunofluorescence staining of MMP2 and MMP9 in FAs with indicated genotype on d28. An uninjured *Tgfbr1* ^*f/f*^ FA was included as a reference for the baseline levels of these proteins. Positive staining of these proteins appears in green. Negative controls omitting the primary antibodies are shown on the far right. White dashed lines delineate EEL. Scale bars, 50 *μ*m. Levels of MMP2 (B) and MMP9 (C) in myointimal and adventitial layers of the indicated FAs (*n* = 7 per genotype). Data were expressed as intensity of the staining (arbitrary unit, AU) normalized to the corresponding region. *P = 0.039 (C), *t‐*test.

An upregulation of metalloproteases, particularly MMP2 and MMP9, has been demonstrated as a driver for vascular restenosis (Tummers et al. 2010; Newby 2012). These enzymes facilitate SMC migration and SMC proliferation at the early stage of myointimal formation and regulate matrix turnover and accumulation during progressive myointimal growth (Newby 2005). To further characterize the phenotype of *Tgfbr1* ^*iko*^ FAs, we compared the production of MMP2 and MMP9 in these FAs with that in *Tgfbr1* ^*f/f*^ FAs 28 days after injury. Consistent with previous reports (Newby and Zaltsman 2000), our assays revealed a constitutive production of MMP2, but modest MMP9 in the tunica media and adventitia of the uninjured FAs. Both MMP2 and MMP9 were present in *Tgfbr1* ^*f/f*^ and *Tgfbr1* ^*iko*^ FAs. A particular pattern of spatial distribution was not identified for MMP2 in either group of FAs. The production of MMP9, however, appeared to be more prominent in myointima than in adventitia of FAs of either genotype (Fig. 7A). Quantitatively, *Tgfbr1* ^*f/f*^ and *Tgfbr1* ^*iko*^ FAs produced a similar amount of MMP2 in the myointima and in the adventitia (Fig. 7B). *Tgfbr1* ^*f/f*^ FAs and *Tgfbr1* ^*iko*^ FAs both displayed a general pattern of higher levels of MMP9 in the myointima than in the adventitia (two‐way repeated measures ANOVA, *P *= 0.015). Further comparisons revealed that *Tgfbr1* ^*iko*^ FAs produced more MMP9 than did *Tgfbr1* ^*f/f*^ FAs in the adventitial layer (*P *= 0.039, *t‐*test; Fig. 7C).

## Discussion

Interventional vascular procedures such as angioplasty and stent placement cause mechanical injury to the target arteries and trigger a “wound healing” process characterized by hyperplastic neointimal thickening and geometric wall remodeling. This repairing process often goes beyond a physiological structural reconstruction, leading to accumulation of excessive neointima and an inward wall remodeling that in turn cause restenosis of the treated arteries. Previous studies have identified TGF*β* as a critical molecular mediator and SMCs as the major cellular effectors in this repairing process (Bobik 2006; DiRenzo et al. 2016). To evaluate the effect of SMC‐specific TGF*β* signaling on regulating NIH and wall remodeling, we deleted SMC TGF*β* type I receptors (i.e., *Tgfbr1* ^*iko*^) and characterized the phenotype of *Tgfbr1* ^*iko*^ FAs following endothelial denudation and intramural dilation. We found that *Tgfbr1* ^*iko*^ attenuated neointimal thickening, and this attenuation was associated with a reduced collagen accumulation. The impact of *Tgfbr1* ^*iko*^ on geometric wall remodeling of the injured FAs was found to be modest. Although these results indicate that *Tgfbr1* ^*iko*^ is beneficial to the arterial patency, other features identified for *Tgfbr1* ^*iko*^ FAs point toward opposite effects, including accelerated cell kinetics, reduced content of *α*‐actin‐producing cells in the myointima, and persistent wall inflammation. These results suggest that *Tgfbr1* ^*iko*^ inhibits early NIH, but the overall phenotype does not favor the long‐term performance of the injured FAs.

Neointimal tissue grows via expanding its cellular population and deposing extracellular matrix. Our results showed that *Tgfbr1* ^*iko*^ FAs formed a thinner neointima but maintained a similar EEL area in comparison with *Tgfbr1* ^*f/f*^ FAs, indicating an inhibitory effect of *Tgfbr1* ^*iko*^ on NIH. Further analysis revealed that the attenuation of neointimal thickening correlated with a greater neointimal cellularity and lower collagen content, suggesting that a reduced matrix accumulation contributes to the inhibitory effect of *Tgfbr1* ^*iko*^ on NIH in the injured FAs. This notion is further supported by the trend of greater MMP9 production in the myointimal layer of *Tgfbr1* ^*iko*^ than in that of *Tgfbr1* ^*f/f*^ FAs. TGF*β* is a profibrotic cytokine that stimulates collagen production in SMCs (Bobik 2006). Despite the fact that this cell autonomous regulation could be a contributor to the reduced collagen content in *Tgfbr1* ^*iko*^ FAs, it is noteworthy that other mechanisms may have contributed to it as well. We have previously shown that the majority of neointimal cells in the FA wire injury model are not derived from the medial SMCs (Yang et al. 2016), which means that only a small portion of neointimal cells in *Tgfbr1* ^*iko*^ FAs were deprived of the ability to synthesize matrix proteins. It is possible that the interaction between cells with and without *Tgfbr1* signaling contributed to the formation of highly cellularized neointima in *Tgfbr1* ^*iko*^ FAs. Using the same model as employed in our study, Kobayashi et al. (2005) reported that global deletion of *Smad3* promotes NIH, and reconstitution of blood cells with wild‐type bone marrow only partially blunted the augmentation, indicating an inhibitory effect of *Smad3* signaling in vascular cells on NIH. Our results suggested a stimulatory effect of SMC TGFBR1 signaling on NIH, which appears to conflict with their study. Although SMAD3 is a signaling intermediate involved in the propagation of TGFBR1 signaling, the signaling network of these mediators does not completely overlap (Massague 2012). In addition, the target cell groups were different (i.e., SMCs vs. entire arterial wall) between their study and ours. These differences may have contributed to the different neointimal responses observed in our experiments.

We have previously shown that the rate of cell proliferation and cell apoptosis in the neointima of experimental bypass vein grafts reaches its peak in the first week following graft implantation and returns toward the baseline level thereafter (Jiang et al. 2004, 2007a). Similar neointimal cell kinetics have also been documented for models of arterial NIH (Kumar and Lindner 1997; Zou et al. 2007). Consistent with these reports, the current study showed that both *Tgfbr1* ^*f/f*^ and *Tgfbr1* ^*iko*^ FAs presented a temporal change in the fraction of BrdU^+^ and TUNEL^+^ cells in the neointimal tissue, with the pattern mirroring that defined in those previous studies. Our results also showed, however, that compared with *Tgfbr1* ^*f/f*^ FAs, the rate of cell proliferation and apoptosis tended to be persistently elevated in *Tgfbr1* ^*iko*^ FAs, indicating that *Tgfbr1* ^*iko*^ drives highly active and dynamic cell kinetics in the injured arteries. We and other groups have previously demonstrated a critical role for SMC‐specific TGF*β* in maintaining the structural homeostasis of the aortic wall (Li et al. 2014; Hu et al. 2015; Schmit et al. 2015). Ablation of SMC TGFBR2 triggers a proliferative response in the aortic media and adventitia (Li et al. 2014; Hu et al. 2015). A remarkable and consistent feature of SMC‐specific TGF*β* deficiency is that the penetrance is limited to aortas at a level above the renal arteries. In the current study, we examined femoral (Fig. 1) and common carotid arteries (data not shown) of *Tgfbr1* ^*iko*^ mice, and evidence of spontaneous pathology was not detected in these arteries. It appears that SMCs disarmed of TGF*β* signaling remain able to maintain structural homeostasis in medium‐sized muscular arteries, but their capacity to handle challenges such as repair of mechanical injury is significantly impaired.

It is generally accepted that medially derived SMCs are the major group of cells that repair structural defects following vascular injury (Nguyen et al. 2013). As the neointimal thickening progresses to a more established stage, the synthetic SMCs reassume their capacity to produce contractile proteins and become quiescent (Gomez and Owens 2012). We therefore compared the production of SM *α*‐actin in *Tgfbr1* ^*f/f*^ and *Tgfbr1* ^*iko*^ FAs. We observed that neointimal lesions forming under conditions without SMC‐TGFBR1 contained fewer *α*‐actin^+^ cells compared to the counterpart wild‐type controls, indicating that the neointimal cells in *Tgfbr1* ^*iko*^ FAs were less differentiated. TGF*β* is a driver for the expression and production of contractile proteins in SMCs (Gomez and Owens 2012). We have previously reported that a fraction of medially derived SMCs, in spite of the intact TGF*β* signaling, is unable to differentiate to regain the ability to produce *α*‐actin in 28‐day‐old FA lesions (Yang et al. 2016). Although ablation of the SMC‐TGFBR1 may have further reduced the number of *α*‐actin‐producing SMCs in the neointima, this cell autonomous mechanism may not fully account for the relatively low content of *α*‐actin^+^ cells in *Tgfbr1* ^*iko*^ FAs, due to the diverse origin of this cell population in neointimal lesions (Zalewski et al. 2002). It is also possible that *Tgfbr1* ^*iko*^ negatively influences the recruitment and differentiation of SMC‐like cells. Although the relationship between the status of neointimal cell differentiation and the long‐term outcome of the injured vessels has not been clearly defined, it seems reasonable that a neointimal lesion consisting of a relatively less differentiated cell population holds a greater risk of acquiring an undesired phenotype as it progresses further to an advanced stage.

An unexpected finding in the current study was the intense inflammation in the adventitial layer of *Tgfbr1* ^*iko*^ FAs, as reflected by more inflammatory infiltrates and a higher level of MMP9 production compared to that of *Tgfbr1* ^*f/f*^ FAs. The impaired neointimal cell differentiation, decreased collagen content, and increased adventitial thickening in *Tgfbr1* ^*iko*^ FAs may also be attributed to the enhanced inflammatory environment in these arteries. Because TGF*β* is a potent immune suppressor (O'Shea and Paul 2010), it is not surprising that global deletion of its downstream mediator *Smad3* triggers inflammatory response in the aortic wall (Ye et al. 2013; Dai et al. 2015). In the current study, deletion of *Tgfbr1* was restricted to medial SMCs. Treatment of SMCs with TGF*β* has been shown to stimulate, rather than inhibit, expression and production of inflammatory mediators (Zhang et al. 2009). It appears that the inflammatory phenotype of *Tgfbr1* ^*iko*^ FAs cannot be fully explained by a loss of the stimulatory effect of TGF*β* on the expression of inflammatory genes in SMCs. Otherwise, it would lead to a conflicting conclusion that both gain and loss of TGF*β* in SMCs are proinflammatory. A well‐known feature of the signaling of TGF*β* and its superfamily members is the promiscuous recognition between receptor members and ligand binding of receptor complexes (Mueller and Nickel 2012). For example, growth and differentiation factor (GDF) 15 stimulates macrophages to express inflammatory cytokines via a TGFBR2‐dependent but TGFBR1‐independent mechanism (de Jager et al. 2011). The same TGFBR2‐dependent GDF15‐signaling mechanism also exists in neurons (Johnen et al. 2007). This promiscuous TGFBR2 signaling mechanism provides a plausible explanation that deletion of *Tgfbr1* triggers an inflammatory response via making more TGFBR2 available to GDF15. Our results therefore favor a hypothesis that the inflammatory mediators produced by *Tgfbr1* ^*iko*^ SMCs recruit inflammatory cells to the adventitial layer, leading to the inflammatory phenotype of the injured *Tgfbr1* ^*iko*^ FAs.

## Conclusions

Our results suggest that deletion of SMC‐specific *Tgfbr1* inhibits NIH in the short term. It causes the injured arteries to express an inflammatory phenotype, however, characterized by less *α*‐actin production, more inflammatory infiltrates, and higher levels of MMP‐9 when compared to *Tgfbr1* ^*f/f*^ controls. Chronic inflammation is a known driver for progressive neointimal thickening. An inflamed neointimal tissue also functions as a “soil” that fosters development of atherosclerosis (Schwartz et al. 1995). Our study thus echoes previous calls for caution because of the potential for side effects from anti‐TGF*β* therapies to interrupt vascular wall homeostasis (Li et al. 2014; Hu et al. 2015; Schmit et al. 2015) and exacerbate certain vascular disease conditions (Wang et al. 2010).

## Conflicts of Interest

The authors declare no conflicts of interest.
